# Genome Informatics and Machine Learning-Based Identification of Antimicrobial Resistance-Encoding Features and Virulence Attributes in Escherichia coli Genomes Representing Globally Prevalent Lineages, Including High-Risk Clonal Complexes

**DOI:** 10.1128/mbio.03796-21

**Published:** 2022-02-15

**Authors:** Sabiha Shaik, Anuradha Singh, Arya Suresh, Niyaz Ahmed

**Affiliations:** a Pathogen Biology Laboratory, Department of Biotechnology and Bioinformatics, University of Hyderabad, Hyderabad, India; Louis Stokes Veterans Affairs Medical Center

**Keywords:** *Escherichia coli*, AMR surveillance, machine learning, virulence, bacterial evolution, genomics, molecular epidemiology, bioinformatics, sequence types

## Abstract

Escherichia coli, a ubiquitous commensal/pathogenic member from the *Enterobacteriaceae* family, accounts for high infection burden, morbidity, and mortality throughout the world. With emerging multidrug resistance (MDR) on a massive scale, E. coli has been listed as one of the Global Antimicrobial Resistance and Use Surveillance System (GLASS) priority pathogens. Understanding the resistance mechanisms and underlying genomic features appears to be of utmost importance to tackle further spread of these multidrug-resistant superbugs. While a few of the globally prevalent sequence types (STs) of E. coli, such as ST131, ST69, ST405, and ST648, have been previously reported to be highly virulent and harboring MDR, there is no clarity if certain ST lineages have a greater propensity to acquire MDR. In this study, large-scale comparative genomics of a total of 5,653 E. coli genomes from 19 ST lineages revealed ST-wide prevalence patterns of genomic features, such as antimicrobial resistance (AMR)-encoding genes/mutations, virulence genes, integrons, and transposons. Interpretation of the importance of these features using a Random Forest Classifier trained with 11,988 genomic features from whole-genome sequence data identified ST-specific or phylogroup-specific signature proteins mostly belonging to different protein superfamilies, including the toxin-antitoxin systems. Our study provides a comprehensive understanding of a myriad of genomic features, ST-specific proteins, and resistance mechanisms entailing different lineages of E. coli at the level of genomes; this could be of significant downstream importance in understanding the mechanisms of AMR, in clinical discovery, in epidemiology, and in devising control strategies.

## INTRODUCTION

Escherichia coli strains, usually described as commensal microbes of the mucosal niche, are also known for their pathogenic acumen, causing a broad spectrum of infections. They have evolved into different pathotypes with the ability to persist in distinct host environments, such as the gut, bloodstream, urinary tract, and meninges (1, 2). Horizontal gene transfer (HGT)-mediated acquisition of mobile genetic elements (pathogenicity islands, phages, plasmids, transposons, integrons, insertion elements, and integrative and conjugative elements), chromosomal reduction (black holes or pseudogenes), and other genomic rearrangements significantly contribute to the evolution of virulence and pathoadaptation in the previously protected niches; this evolves and facilitates some of the novel pathogenic lineages and their successful global dissemination (3–5). The emergence of highly virulent and antimicrobial-resistant pathogens poses a serious threat to global public health systems, and the lack of novel or efficient drugs in the clinical development pipelines could result in increased morbidity, mortality, and associated medical expenses (6, 7).

With the continuous emergence and dissemination of multidrug-resistant (MDR) bacteria, the acquisition of resistance also has been associated with other fitness advantages, such as enhanced virulence (8, 9). To allow the understanding of the evolutionary dynamics of E. coli, several classification methods have evolved that categorized E. coli genomes into different pathotypes on the basis of host or organ tropism (extraintestinal pathogenic E. coli [ExPEC], intestinal pathogenic E. coli [IPEC], uropathogenic E. coli [UPEC], and avian pathogenic E. coli [APEC]), disease conditions (diarrhea-associated hemolytic E. coli [DHEC] and enterohemorrhagic E. coli [EHEC]), and virulence factors (Shiga toxin-producing E. coli [STEC]) (10). However, the emergence of hybrid pathotypes, such as ST141, limited the usefulness of this conventional system of classification (11). Another approach suggests dividing E. coli genomes into phylogroups such as A, B1, B2, and D using multilocus enzyme electrophoresis or by triplex PCR using a combination of *chuA* and *yjaA* genes along with the TSPE4.C2 DNA fragment (12) or on the basis of *in silico* whole-genome data (13). Among these classification methods, multilocus sequence typing (MLST), which identifies clonal groups using the allelic distribution of seven housekeeping genes, is now considered the gold standard to understand the evolution and epidemiology of pathogenic lineages of bacteria (14).

Among different sequence types (STs) in E. coli, the ESBL CTX-M-15-producing ExPEC lineage ST131, with its virulence capabilities and MDR phenotype, has been the target of several studies for over a decade (15). The evolution of ST131 as a pathogenic lineage has been linked with the loss of chromosomal content, genetic exchange, and coevolution with antimicrobial resistance (AMR) gene repertoire (16). Several studies have explored the clonal distribution of STs, which were MDR, but relatively few have addressed how drug-susceptible STs are distributed. In a study conducted by Adams-Sapper et al. (17), both MDR clones (ST131 and ST69) and antibiotic-susceptible strains (ST73 and ST95) were observed in bloodstream infections (17). In addition to ST131, ST405 is another clone known to be associated with the worldwide distribution of *bla*_CTX-M-15_ on IncFII plasmids (18). There is still much to understand regarding the factors that lead to the success of drug-susceptible as well as multidrug-resistant lineages (19).

Advancements in sequencing technologies, in addition to the reduced costs, have provided a boost to rapid evolution of the bacterial genomics realm over the past decade, with an increased focus on population-level sequencing studies. Large-scale genome data of pathogenic bacteria from several such studies could be an invaluable resource to gain insights into the evolutionary and ecological trends of the emergence of a pathogenic microorganism (20–23). Inferences from large-scale, high-resolution epidemiological genomic data could address questions pertaining to evolutionary origins, transmission dynamics, and outbreak patterns of bacteria with AMR (24, 25). The in-depth resolution offered by large-scale genome data analyses could help track the frequencies of pathogenic variants, genotype shifts, and prevalence (26) and could provide valuable insights into the underlying gene loss/acquisition in a particular subpopulation. In addition, these insights could further help to understand various factors that influence better fitness and survival of specific subpopulations (26, 27).

With a surge in the availability of genome sequence data, there is a need to upgrade the conventional genome data analysis pipelines using more powerful and advanced computational methodologies for mining relevant information (28). The amount of data available for comparative genomics alone was found to be on par with other big data generators, such as astronomy, YouTube, and Twitter, as discussed previously (29). With the continuous increase in the dimensionality of genomics data, machine learning approaches such as supervised and unsupervised learning are proving to be increasingly robust, reliable, and computationally efficient for comparative genomics. Machine learning algorithms help not only in classification or regression-related objectives but also in the selection of important relevant features for inference (28). In this study, we have extensively deciphered a data-driven, feature selection approach using machine learning to identify and understand the genomic variations (genes/mutations/other elements) in E. coli. These features *a priori* stratify the strains/genomes of the 19 different ST lineages of E. coli in the context of fitness advantages that potentially lead to phenotypic AMR and virulence properties.

## RESULTS

### Curated data sets for comparative genomic analyses.

After data curation, a total of 5,653 genomes belonging to 19 different STs were selected for comparison. Only STs with a minimum of 100 genomes were considered for better statistical relevance. ST131 was observed to represent the highest number of genomes, i.e., 1,101 genomes, followed by ST10 (1,036 genomes), while the remaining STs represented genome numbers ranging from 105 to 543 (Table 1). More than 98% of genomes from the STs were associated with a single phylogroup (see File S1 at https://github.com/Sabiha-NGS/EcoliGenomics2021).

**TABLE 1:** Serotype information of 19 STs considered in the study

ST	No. of genomes	Total no. of serotypes	Major two serotypes (%)
ST131	1,101	13	O25:H4 (84.5), O16:H5 (8.9)
ST10	1,036	222	O16:H48 (26.0), O89:H9 (4.1)
ST11	543	3	O157:H7 (99.1), −:H7 (0.6)
ST95	418	13	O1:H7 (27.8), O18:H7 (21.8)
ST73	318	10	O6:H1 (59.7), O2/O50:H1 (11.0)
ST69	276	25	O77/O17/O44/O106/O73:H18 (32.2), O77/O17/O44/O106:H18 (21.7)
ST58	222	79	O8:H25 (10.8), −:H21 (6.8)
ST410	214	24	O8:H9 (42.5), −:H9 (24.3)
ST101	202	68	−:H31 (8.4), O82:H8 (7.4)
ST38	164	26	O86:H18 (30.5), O2/O50:H30 (10.4)
ST155	162	61	−:H21 (11.7), O86:H51 (6.2)
ST167	150	12	O89:H9 (68.7), O89:H5 (6.7)
ST117	142	34	O24:H4 (23.2), O33:H4 (9.9)
ST405	134	5	O102:H6 (92.5), O2/O50:H4 (3.7)
ST48	126	57	−:H11 (8.7), O10:H5 (7.1)
ST648	121	22	O1:H6 (50.4), O153:H6 (8.3)
ST127	112	4	O6:H31 (92.9), −:H31 (4.5)
ST156	107	50	−:H28 (13.1), −:H25 (9.3)
ST12	105	7	O4:H5 (63.8), O4:H1 (17.1)

### Prevalent serotypes and plasmid incompatibility groups among the 19 STs.

The 5,653 genomes analyzed in this study contained 579 different serotypes (Table 1), with a large number of serotypes present in the majority of STs. Among the 1,036 ST10 genomes, there were 222 different serotypes, with O16:H48 (26%) being the most prominent serotype. Similarly, ST58 (222 genomes) belonged to 79 different serotypes, whereas ST101 (202 genomes) encompassed 68 different serotypes. In contrast, ST131, ST11, ST73, ST405, and ST127 were observed to be prominently associated with one major serotype. In ST11, 99.1% of the 543 genomes were from the O157:H7 serotype alone. ST131, ST73, ST405, and ST127 were also associated mostly with serotypes O25:H4 (84.5%), O6:H1 (59.7%), O102:H6 (92.5%), and O6:H31 (92.9%), respectively (Table 1).

Among the plasmid classes in the 19 STs, it was observed that the replicon types of IncF class were predominantly present. IncFII replicon type was found in higher prevalence among genomes of ST131 (87.64%), ST11 (95.94%), ST95 (78.46%), ST69 (78.26%), ST410 (80.84%), ST167 (76.66%), and ST405 (91.04%). IncFIA also was found in the majority of the genomes of ST131 (80.01%) and ST410 (74.29%), whereas IncFIB was found to be highly prevalent among genomes of ST131 (74.47%), ST11 (96.68%), ST95 (91.86%), ST410 (90.18%), ST101 (73.26%), ST117 (90.14%), ST405 (74.62%), and ST648 (74.38%). Other incompatibility groups, such as IncX1, Incl1-I, IncQ1, and IncY, were found among a few genomes from different STs (see Fig. S1 at https://github.com/Sabiha-NGS/EcoliGenomics2021).

### Understanding the resistome across the 19 STs of E. coli.

**FIG 1:**
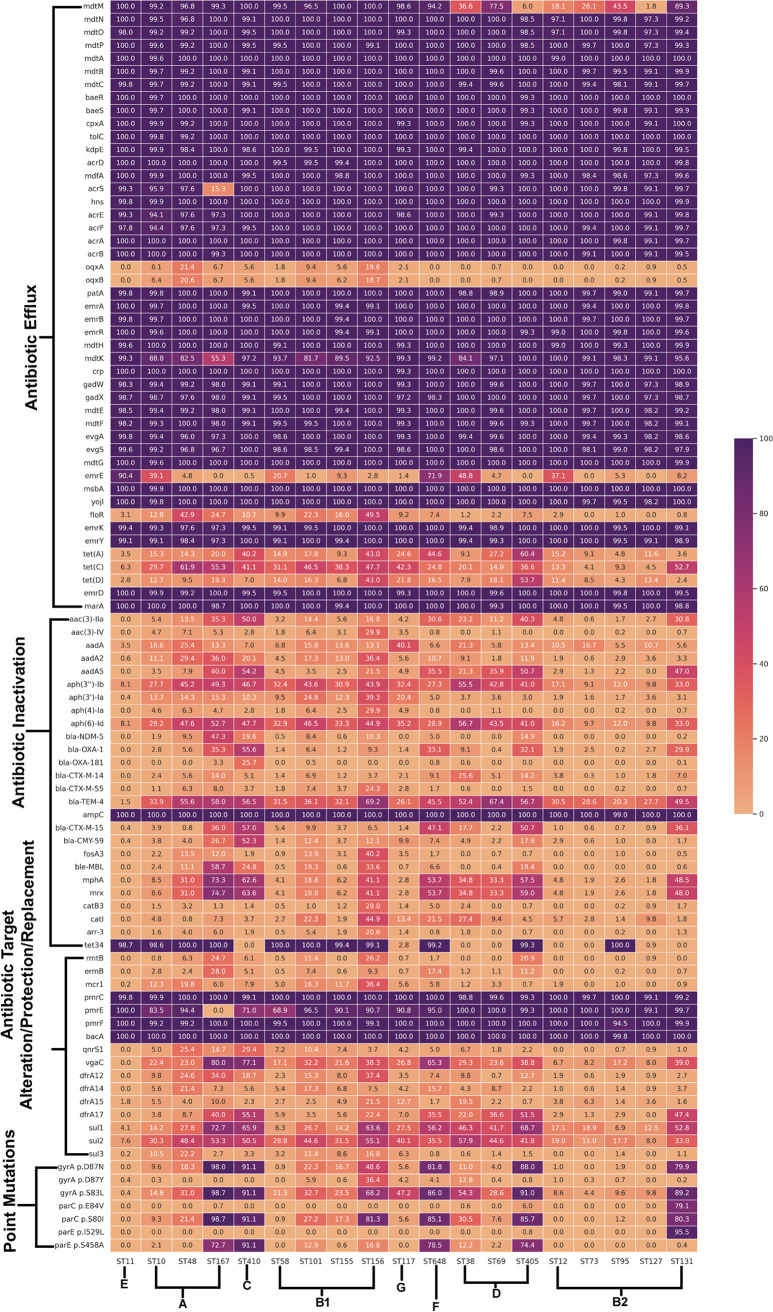
Heat map depicting the resistome profiles of the 5,653 genomes from 19 different STs. Gene names are represented on the *y* axis and the ST lineage on the *x* axis. The % presence of each of these genes at the ST level was calculated using the formula (presence in no. of genomes of ST/total no. of genomes in ST) × 100 and plotted using the matplotlib module. The color key represents % presence.

Resistome screening of 5,653 E. coli genomes revealed 247 AMR genes. Among them, 89 were found to be present in at least 20% of the genomes of any other STs (Fig. 1; see File S2 at https://github.com/Sabiha-NGS/EcoliGenomics2021). Efflux pump-encoding genes were observed to have a similar prevalence pattern among all the STs considered in the study, except for a few other genes, such as *mdtM*, *oqxA*, *oqxB*, *mdtK*, *emrE*, *floR*, *tet*(A), *tet*(C), and *tet*(D). *mdtM* was found to be present in less than 30% of the genomes from each of ST12, ST127, ST405, and ST73. While a majority of the genomes from ST11 (90.4%) and ST648 (71.9%) harbored *emrE*, a lesser prevalence of the same was observed in ST131 (8.2%) and other STs (Fig. 1).

Multiple genes [*aac(3)-IIa*, *aac(3)-IV*, *aadA*, *aadA2*, *aadA5*, *aph(3′')-Ib*, *aph(3′)-Ia*, *aph(4)-Ia*, and *aph(6)-Id*] encoding different variants of aminoglycoside transferase enzymes were observed to be less prevalent (<20%) across ST11, ST127, and ST73. Three different genes, namely, *sul1*, *sul2*, and *sul3*, encoding resistance against the sulfonamide class of drugs, were also compared. Both *sul1* and *sul2* genes were present in larger numbers among different STs than the *sul3* gene, which was missing among genomes belonging to ST12, ST127, and ST73.

Among the β-lactamase-encoding genes, the *ampC* gene was observed to be most prevalent across different STs (>99%), followed by *bla*_TEM-4_. No ST-linked prevalence pattern was observed in the case of the other β-lactamase-encoding genomes. *qnrS1*, which encodes resistance to the fluoroquinolone class of drugs, was present in 25.4% and 29.4% genomes of ST48 and ST410, respectively, while in other STs this particular gene was less prevalent (<15%). Among the point mutations identified by PointFinder, those occurring more frequently were observed in genes encoding subunits of class II topoisomerase enzymes (*gyrA*, *parC*, and *parE*). *gyrA* was observed to be most frequently mutated at positions 83 (S83L) and 87 (D87N). ST131 displayed two unique mutations in *parC* (E84V) and *parE* (I529L), which were not shared by the other STs.

### Virulome analysis.

**FIG 2:**
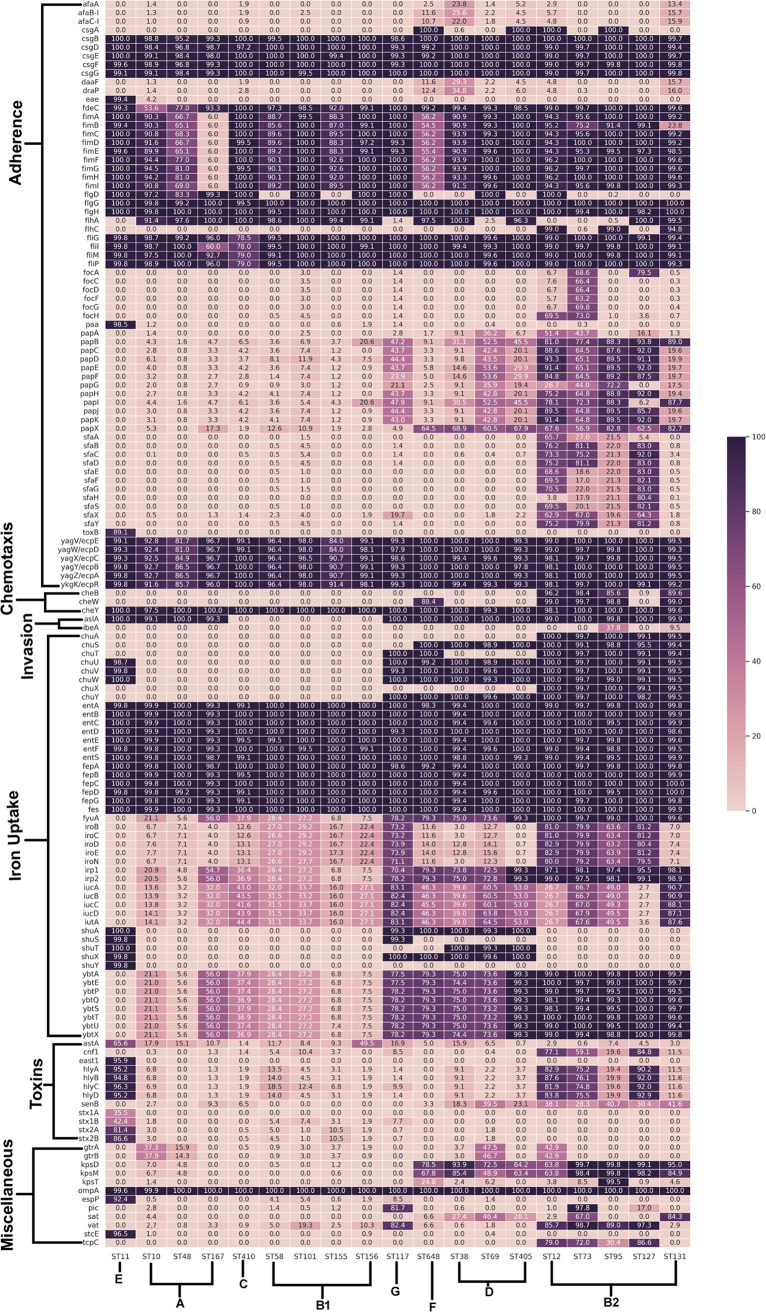
Heat map depicting the virulome profile of the 5,653 genomes from 19 different STs. Gene names are given on the *y* axis and the ST lineage on the *x* axis. The % presence of each of these genes at the ST level was calculated using the formula (presence in no. of genomes of ST/total no. of genomes in ST) × 100. The color key represents % presence.

**FIG 3:**
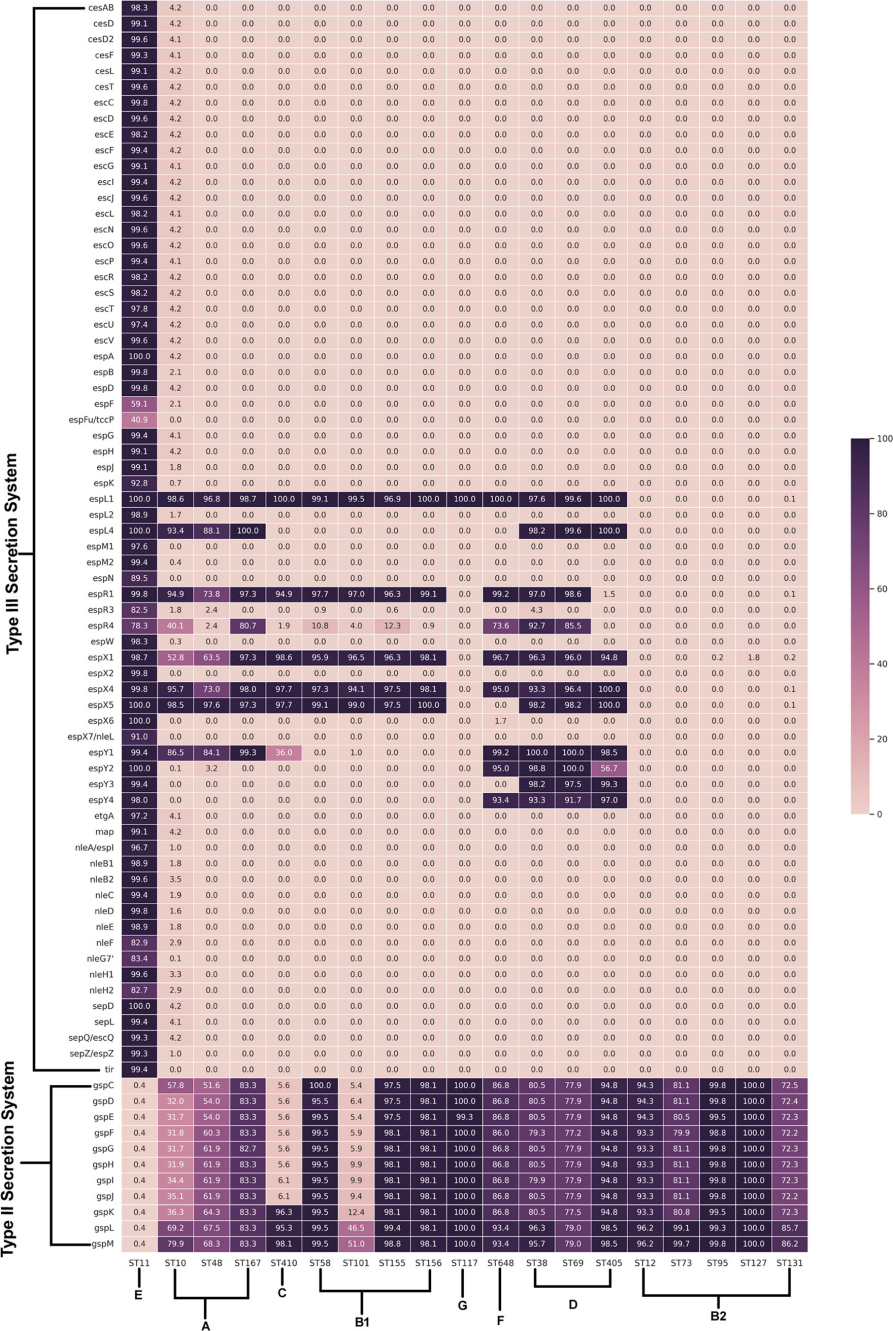
Heat map depicting the prevalence of genes linked with secretion systems in the 5,653 genomes from 19 different STs. Gene names are mentioned on the *y* axis and the ST lineage on the *x* axis. The % presence of each of these genes at the ST level was calculated using the formula (presence in no. of genomes of ST/total no. of genomes in ST) × 100. The color key represents % presence.

The entire data set was screened for the virulence factors associated with pathogenic potentials in E. coli (see File S3 at https://github.com/Sabiha-NGS/EcoliGenomics2021). Multiple virulence factors, such as adhesins, invasins, iron uptake systems, secretion systems, and transporters, were observed in abundance, as depicted in Fig. 2 and 3. Among adhesion genes, *csgBDEFG* associated with curli fiber production, *fdeC* that encodes the attaching and effacing protein, *fimABCDEFGHI* codifying type I fimbriae, flagellar genes (*flgGH*, *fliGIMP*), and *yagVWXYZK-ecpEDCBAR* genes encoding E. coli pili were prevalent (>50%) across all STs except ST167 (type I fimbrial protein-encoding genes were present in only 6% of the genomes) and ST131 (*fimB*; the type I fimbria-encoding gene was present in 23.7% of genomes). It was observed that the *csgA* gene involved in curli fiber production was present in all the genomes belonging to ST12, ST405, ST648, and ST95 but was entirely absent from the rest of the STs except ST38 (0.6%).

F1C fimbria-encoding genes such as *focACDFGH* were uniquely prevalent in the genomes of ST73. In addition, we observed that three adhesin-encoding genes (*eae*, *paa*, and *toxB*) were highly prevalent in ST11 genomes but were less prevalent across the rest of the STs. The flagellar transcriptional regulator *flhC* was observed to be exclusively present only in the genomes of ST131, ST12, and ST95. We also observed that genomes from phylogroup B2 were highly abundant in genes encoding P and S fimbriae, such as the *pap* and *sfa* gene clusters. Genes encoding chemotaxis proteins (*cheBWY*), invasion proteins (*aslA* and *ibeA*), heme uptake (*chuASTUVWXY* and *shuASTXY*), enterobactin synthase (*entABCDEFS*), ferrienterobactin transporter (*fepABCDG*), yersiniabactin siderophore (*fvuA*, *irp12*, and *ybtAEPQSTUX*), salmochelin siderophore (*iroB*, *iroC*, *iroD*, *iroE*, and *iroN*), and aerobactin siderophore (*iutABCD* and *iucA*), observed within the genomes of different STs, were either present in more than 80% of the strains of a specific ST or completely absent from another ST lineage. It was observed that genomes belonging to ST11 showed a complete absence of *cheBW*, *ibeA*, *chuASTXY*, *fyuA*, *iroBCDEN*, *irp12*, *iucABCD*, *iutA*, and *ybtAEPQSTUX* genes (Fig. 2).

Genes encoding different toxin proteins, such as alpha-hemolysin (*hlyABCD*), Shiga-like toxin (*stx1A*, *stx1B*, *stx2A*, and *stx2B*), and enterotoxins like *senB* were widely distributed among the ST11, ST12, ST127, and ST73 genomes. Moreover, ST11 was the only group found to be positive for *east1* (enteroaggregative heat-stable enterotoxin 1) and *stx1* (Shiga toxin) genes. In addition to these genes, cytotoxic necrotizing factor-encoding gene *cnf-1* was also prevalent (>55%) in ST12, ST127, and ST73.

Genes encoding type III secretion systems (locus of enterocyte effacement [LEE]) and their effectors (non-LEE encoded), type II secretion systems, were also predicted by VFDB (Fig. 3). It was observed that a majority of the genes encoding type III secretion systems predominantly observed in the strains of ST11 were completely missing from the other lineages. Other type III secretion system-encoding genes (*espL1*, *espL4*, *espR1*, *espX1*, *espX4*, *espX5*, *espY1*, *espY2*, and *espY3*) were observed to be present in other lineages except ST131, ST12, ST73, ST127, and ST95, belonging to the phylogroup B2. Genes encoding type II secretion systems were found to be missing from ST11 genomes.

### Integrons and transposons.

**FIG 4:**
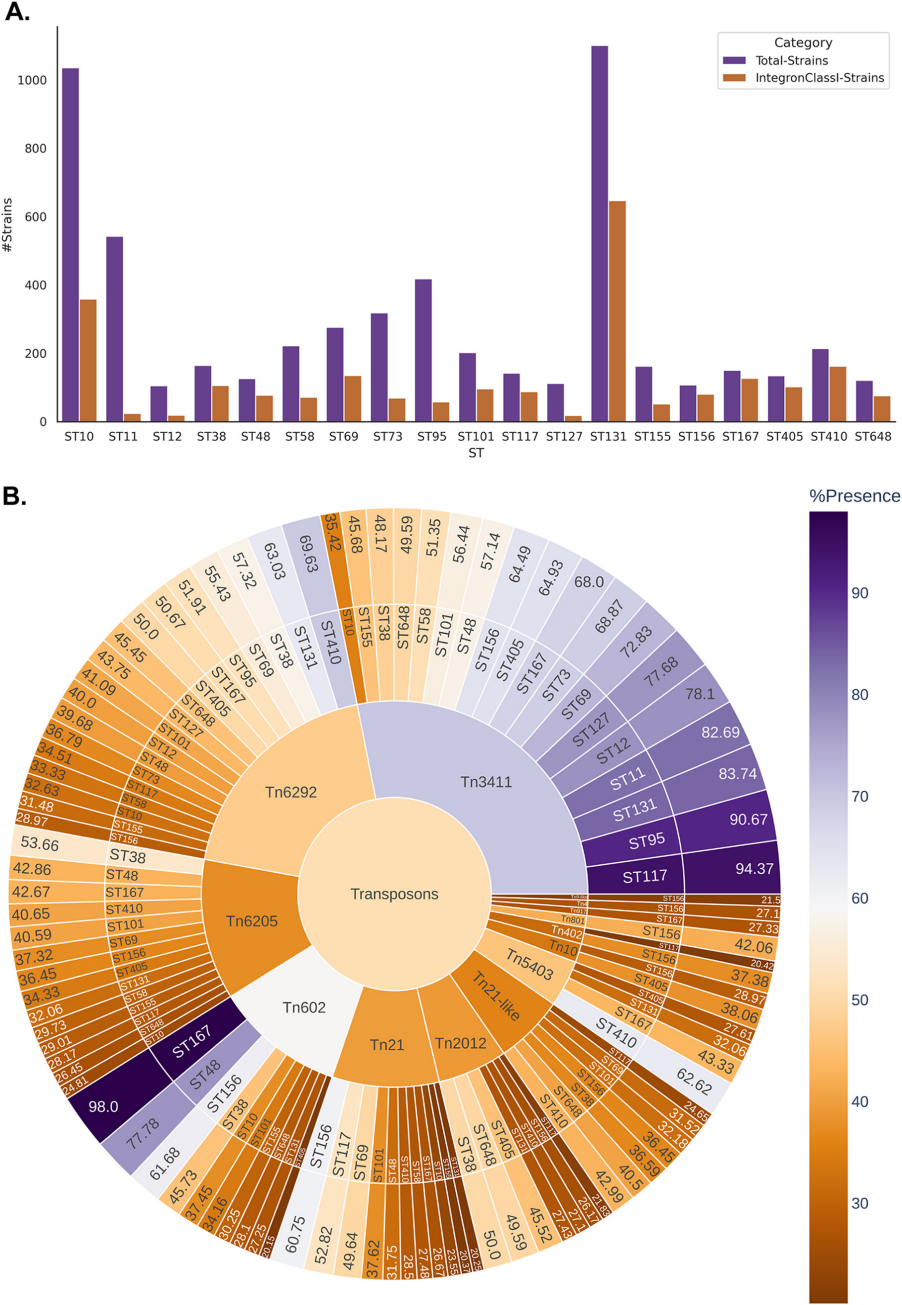
(A) Bar plot depicting the number of genomes harboring class 1 integrons among the 19 STs considered under this study. (B) Sunburst plot depicting the prevalence of transposons among the 19 STs.

Integrons belonging to classes 1, 2, 3, and 4 were predicted to be present among the genomes from 19 STs in this study. Among these four classes, class 1 integrons were observed to be highly prevalent among the ST167 (84.6%), ST405 (76.1%), ST410 (75.7%), and ST156 (74.7%) lineages (see File S4 at https://github.com/Sabiha-NGS/EcoliGenomics2021), although they were present in only minor proportions in the genomes of ST11 (4.4%), ST12 (18.09%), ST95 (13.8%), and ST127 (16.07%) (Fig. 4A). The class 2, 3, and 4 integrons were only scarcely distributed among the 19 STs (File S4).

Forty-eight different types of transposons were present in the genomes analyzed by us (File S4). Among these, Tn*3411* and Tn*6292* transposons were most prevalent in the genomes across most STs (Fig. 4B). Only 2% of the ST11 genomes were observed to harbor Tn*6292*, unlike other STs, wherein the prevalence was more than 25%. Tn*602* was observed to be present in more than 60% of the genomes belonging to ST156 (61.68%), ST167 (98%), and ST48 (77.78%) (Fig. 4B). Overall, ST156 was observed to harbor a wide diversity of transposons. Although Tn*3411* was highly prevalent in ST11 (82.7%), other transposon classes were observed to be present in less than 10% of the genomes of this ST (Fig. 4B).

### Pangenome content of 5,653 genomes from 19 STs.

A total of 137,919 orthologous groups of genes were identified by the software Roary, of which 1,498, 5,517, and 130,870 genes constituted the soft core genes (95% ≤ genomes < 100%), shell genes (15% ≤ genomes < 95%), and cloud genes (0% ≤ genomes < 15%), respectively.

### Phylogroup- and ST-specific clustering observed in the 5,653 genomes.

**FIG 5:**
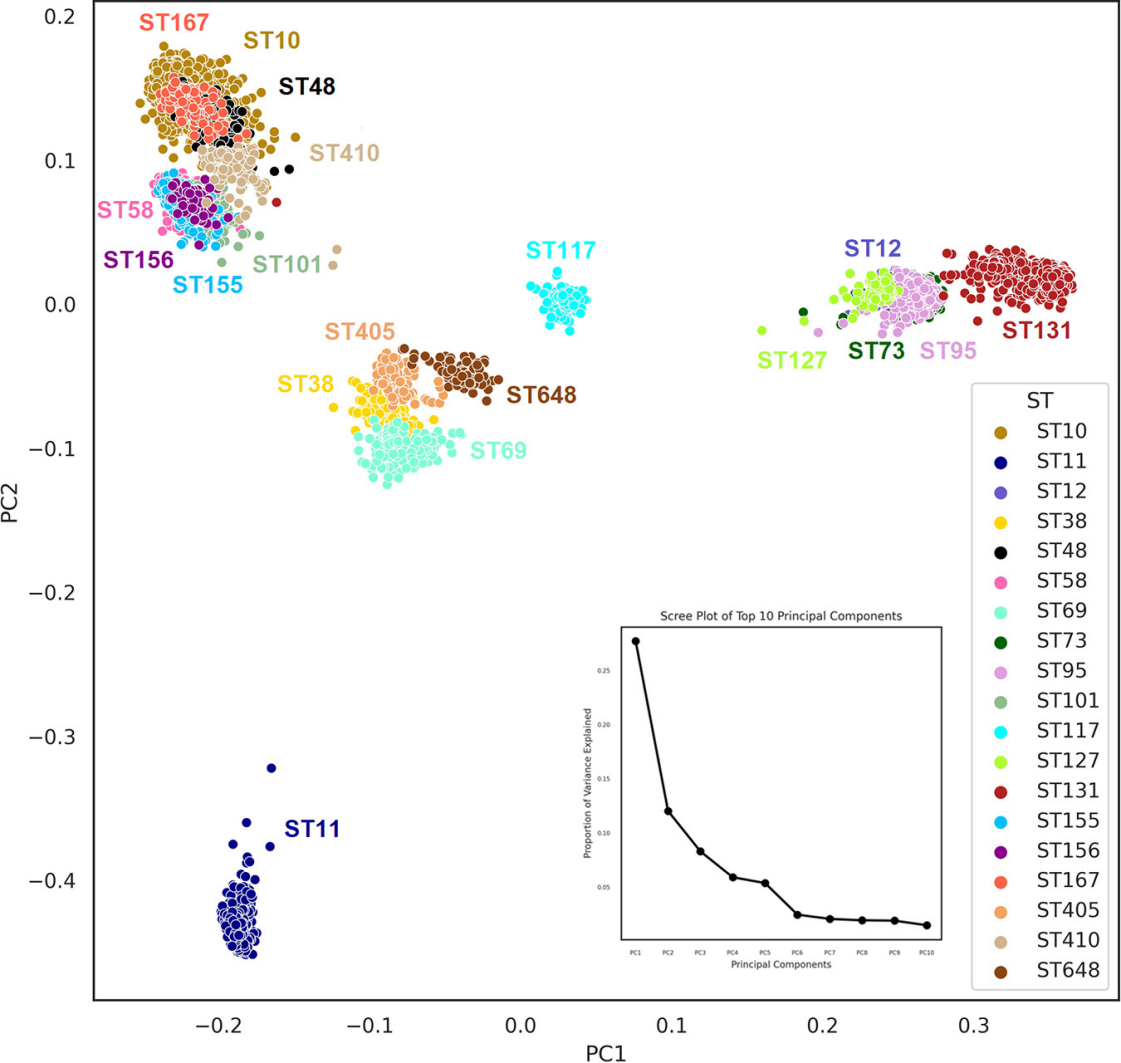
Principal coordinate analysis (PCoA) plot (with the top 2 PCoA axes) showing the clustering of genomes from different STs. A nested scree plot within the image depicts the proportion of variance explained by the top 10 principal components (PCs).

Principal coordinate analysis (PCoA), a method of multivariate analysis, can help in identifying the proximities of the genomes based on a distance matrix. PCoA based on the distances (Jaccard matrix) between the genomes using the genomic features revealed distinct clustering of ST-specific genomes. Clustering of STs with respect to their phylogroups also was observed in the scatterplot of the top two ordination axes of PCoA (Fig. 5). ST167, ST10, and ST48 from phylogroup A, ST410 from phylogroup C, and ST58, ST156, ST155, and ST101 from phylogroup B1 were seen in close proximity. Close proximities between STs from the same phylogroup in the case of phylogroup D (ST405, ST69, and ST38) and phylogroup B2 (ST131, ST95, ST73, ST127, and ST12) were observed. ST11 (phylogroup E) was observed to be placed distantly from all other STs. Further, permutational analysis of variance (PERMANOVA) of the Jaccard distance matrix with a *P* value of 0.001 has confirmed the significant role of our selected features in capturing the genomic variations among the 19 clonal lineages (STs). Chi-square test, to calculate a significant correlation between two categorical variables, was used to test the correlation of the 11,988 features with the target ST classes. On the whole, the 11,578 features selected by us were observed to have a significant correlation (≤0.005) with the target ST column.

### Deriving feature importance from RF classifier.

Classification algorithms are a part of supervised machine learning techniques that can predict the class of a new observation after being trained. The Random Forest (RF) Classifier and Extra Trees Classifier were the best performing algorithms among the five classification methods based on performance metrics such as accuracy, precision, recall, and F1 score (Table 2). Since the RF method could also list out the importance of the features, it was selected for further hypertuning using a set of optimal hyperparameters to identify the best parameters to train. The learning curve of the RF classifier with the best parameters depicted that the training score was around the maximum, and the validation score could be further improved by increasing the quantum of the training data (see Fig. S2 at https://github.com/Sabiha-NGS/EcoliGenomics2021). Only 435 important features from the best RF model, the elimination of which led to a significant drop in accuracy, were selected for further analysis.

**TABLE 2:** Performance of five supervised algorithms that support multiclass classification

Algorithm	Training accuracy	Testing accuracy	F1 score	Recall	Precision
RandomForestClassifier	1.000000	0.999410	0.999408	0.999410	0.999412
ExtraTreesClassifier	1.000000	0.999410	0.999408	0.999410	0.999412
KNeighborsClassifier	0.998989	0.998231	0.998227	0.998231	0.998250
CategoricalNB	0.995198	0.998231	0.998242	0.998231	0.998288
XGBoost	0.183220	0.183373	0.016311	0.052632	0.009651

The importance of these 435 features was further cross-validated using a subsampling method of the same training and testing data sets. Cross-validation of the three KNN models with the same training and testing data sets but with different feature lists revealed that the model with only 435 selected features performed best with a testing accuracy of 1. While the model with all 11,988 features showed a testing accuracy of 0.998, the one without 435 features showed a relatively lower accuracy score of 0.997 (File S5 at https://github.com/Sabiha-NGS/EcoliGenomics2021).

### Significant genomic features associated with 19 clonal lineages of E. coli.

**FIG 6:**
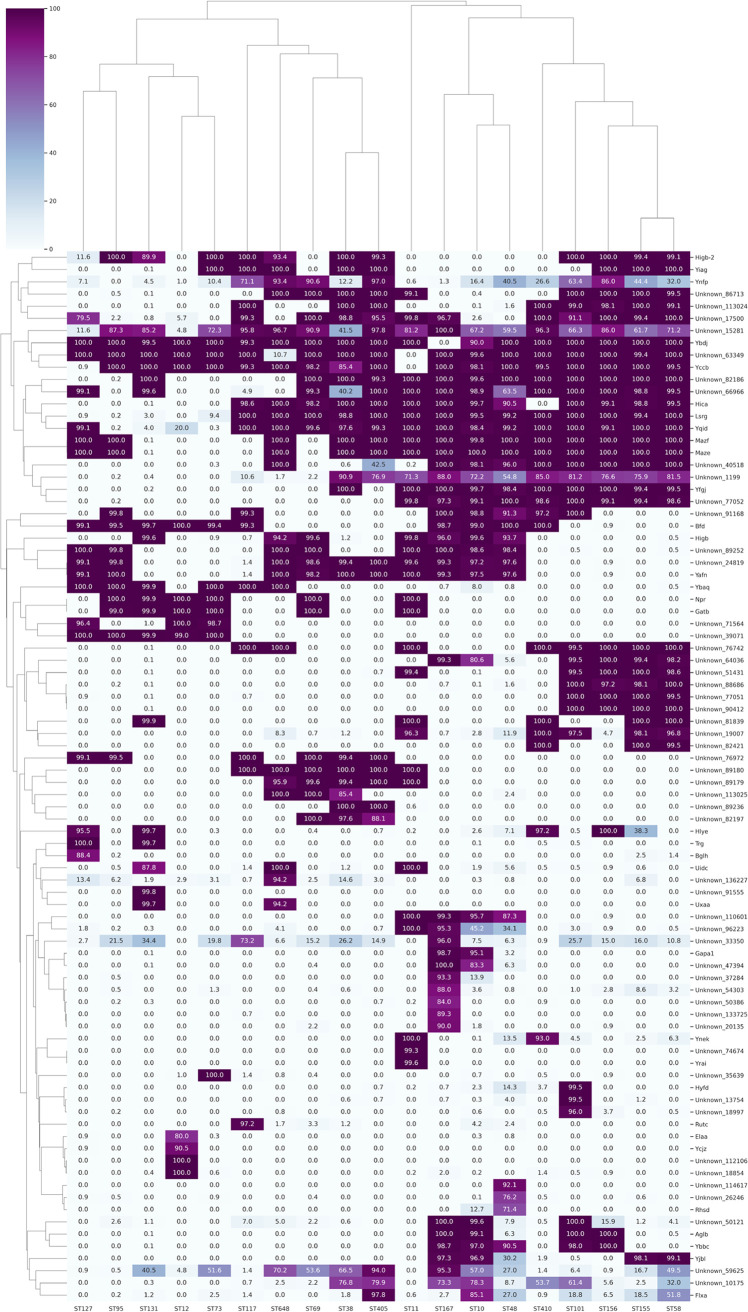
Cluster map depicting the prevalence of validated important features from the RF model among the 19 STs considered under this study. The % presence of each of the proteins at the ST level was calculated using the formula (presence in no. of genomes of ST/total no. of genomes in ST) × 100. Feature names are represented on the *y* axis and the ST lineages on the *x* axis. The color key represents % presence.

Further validation of the protein sequences of these 435 genomic features using DIAMOND (30) in the entire data set has shown that 86 proteins were found to have ST-linked differential patterns (Fig. 6).

A few proteins were observed to be predominantly missing from only one or two STs, such as Ybdj (ST167), Unknown_63349 (ST11, ST648), and Yccb (ST11 and ST127). There were also a few proteins that were exclusively present in one or two STs such as FlxA (ST10 and ST405), RutC(ST117), RhsD, and a few others. A cluster of proteins was observed to be exclusively present in a single ST such as ST48 (Unknown_6246 and Unknown_14617), ST12 (ElaA, Ycjz, Unknown_12106, and Unknown_8854), ST101 (HyfD, Unknown_3754, and Unknown_8997), ST11 (YraI and Unknown_74674), and ST167 (Unknown_37248, Unknown_54303, Unknown_50386, Unknown_133725, and Unknown_20135). Toxin-antitoxin proteins MazF-MazE were found to be exclusively missing from genomes of ST131, ST12, ST73, ST117, and ST69 but were present in all the genomes of the remaining STs.

A cluster map of the aforementioned 86 proteins plotted with the percentages of presence has shown phylogroup-based clustering of these STs (Fig. 6). Protein Bfd with CDD domain bacterioferritin-associated ferredoxin was observed to be exclusively present in phylogroups B1, E, D, and F. A hypothetical protein, Unknown_76742, was also seen only in phylogroups B1, C, E, F, and G.

Annotation of these 86 proteins has revealed that a majority of them (variously) harbored conserved domain families of toxins, antitoxins, nudix hydrolase, type III secretion system, HNH nucleases, antibiotic synthesis monooxygenase domain, N-acetyltransferase, helix-turn-helix domains, outer membrane porins, and other features of unknown function (see File S6 at https://github.com/Sabiha-NGS/EcoliGenomics2021).

## DISCUSSION

E. coli isolates, with their enormous diversity in terms of clonal lineages, are known for transitioning between commensalism, mutualism, and pathogenicity depending on the selection pressures and niche-specific constraints or adaptations experienced by the genomes (10). With the rise in AMR among the pathogenic strains of E. coli, it is listed with the top five priority pathogens by the WHO (31). With no straightforward distinction between commensal and pathogenic groups, it is important to understand the associations between the virulence factors, resistance mechanisms, and other pathogenic coordinates along with niche-related information entailing different sequence types, lineages, pathotypes, and phylogroups to design control strategies for these superbugs (10).

With a curated data set of 5,653 E. coli genomes, we attempted to identify and understand the involvement of different genomic features with a plausible role in the evolution of 19 different ST lineages with varied pathogenic, resistance, and fitness capabilities, some bearing high risk of transmission with pandemic potentials. ST10 from our study has shown a wide serotype diversity, wherein its 1,036 genomes belonged to 222 serotypes; this is in agreement with a previous report (32). While a majority of the sequence types revealed a very high antigenic diversity, a few STs, such as ST131, ST11, ST405, and ST127, showed limited antigenic diversity where more than 80% of the genomes belonging to these STs represented a single serotype.

A majority of the 543 genomes of ST11 were from the serotype O157:H7, which was previously reported to be associated with highly pathogenic STEC (33). ST11 was also found to be one of the most intriguing sequence types with a unique virulome profile, unlike other STs. ST11, the only representative of phylogroup E, from our study, was also observed to form a distinct cluster in the scatterplot of the top two axes of PCoA (Fig. 5) while also revealing peculiar trends concerning resistome (Fig. 1) and virulome (Fig. 2 and 3) profiles. The StcE protease, previously reported to help in the adherence of EHEC serotype O157:H7 to host cells, is secreted by the pO157-encoded *etp* type II secretion system (34). This gene was seen in 96.5% of the ST11 genomes, but was absent from the majority of the STs. EHEC isolates belonging to O157:H7 adhere to epithelial cells and form pedestals with the help of LEE-encoded proteins, including EspA, EspB, EspD, Tir, and intimin (35). Genes encoding such proteins as *espABD*, *tir*, and *eae* were highly prevalent among the isolates of ST11 in our study. Other EHEC-associated coordinates, such as *eae*, toxin *toxB*, and type III secretion system genes (36), were observed to be highly prevalent in the genomes of ST11 alone. On the other hand, the type II secretion system-linked genes encoding pseudopilus (*gspG*, *gspI*, *gspK*, and *gspH*), outer membrane complex (*gspD*), inner membrane platform (*gspC*, *gspF*, *gspL*, and *gspM*), and cytoplasmic ATPase (*gspE*) (37) were found to be highly prevalent among all STs other than ST11. The type II secretion system, which is found in a subset of Gram-negative bacteria irrespective of their pathogenicity, has been known to help in their adaptation in ecologically diverse niches (38).

ST131, with a major serotype, O25:H4, belonging to phylogroup B2, as observed in our study, was previously linked with the presence of genes encoding CTX-M enzyme and tetracycline resistance-associated genes, such as *intl1*, and gene cassettes, such as *dfrA12-aadA2* and *dfrA17-aadA5* (39). Results from our study demonstrated that ST131 harbors a majority of the AMR-associated genes and mutations as well as displays a significant virulence profile. Previous studies demonstrated that the epidemiological success of ST131 cannot be attributed to a single factor and is one of the most important STs associated with multilayered fitness advantages that could be a result of enhanced virulence potential, AMR, metabolic activity, and some yet-to-be-revealed evolutionary mechanisms (40).

Efflux pumps form an integral part of the bacterial intrinsic resistome, and most of them are part of the core genome (41, 42); thus, a huge repertoire of genes encoding efflux pumps and their regulators were observed in almost all the genomes included in the study. The *mdtM* gene linked with intrinsic resistance against chloramphenicol, ethidium bromide, alkaline pH homeostasis, and quaternary ammonium compounds (43–45) displayed a varied degree of prevalence among different STs in our study.

Class 1 integrons, which were previously reported to play an important role in the spread of AMR genes among the Gram-negative pathogens (46), were observed to be present in the majority of the genomes from all STs represented in our data set. The presence of different tetracycline resistance genes in E. coli was reported to be positively correlated with the carriage of class 1 integrons among the commensal E. coli isolates (47). ST405 and ST156, from different phylogroups, showed comparable prevalence rates of the tetracycline resistance-linked *tet*(A), *tet*(C), and *tet*(D) genes as well as class 1 integrons. A low prevalence of *tet* genes and class 1 integrons was observed in STs such as ST73, ST127, ST12, ST95, and ST11.

*floR*, an MDR efflux family gene encoding combined resistance against florfenicol and chloramphenicol, previously reported to be prevalent in cattle and poultry (48, 49), was observed in ST156 (49.5%) and ST48 (42.9%) genomes. *emrE*, highly prevalent in ST11 and ST648 as observed herein, encodes a small multidrug transporter causing resistance against quaternary cation compounds (50). The resistance against the sulfonamide class of drugs is via *de novo* mutations in the DHPS (*folP*) gene or by the acquisition of alternative DHPS genes (*sul1*) (51). *sul1* and *sul2* genes were observed in all the genomes of ST167, ST410, ST405, ST38, ST131, ST69, ST648, and ST156.

Invasion-linked *aslA* (bacterial arylsulfatase gene), which was previously reported to help E. coli K1 in the invasion of the blood-brain barrier (52), was seen to be present in almost all the genomes from phylogroups A, B2, D (except ST101), E, G, and F. These genes were missing in the genomes from ST155, ST58, and ST156 (phylogroup B1) as well as from ST410 (phylogroup C) and ST101 (phylogroup D). However, the *ibeA* invasin gene, with a significant role in invasion by AIEC (adherent-invasive E. coli) pathogroups, was seen to be present in ST95 (37.8%) and absent from all other STs except ST131 (9.5%) (53). ST131, ST73, and ST69, which are known to be ExPEC isolates, were previously observed to be prevalent in bloodstream infections (54). Genomes from these STs were observed to show a moderately high prevalence of adhesins, iron acquisition factors, protectins, and toxins. Type 1 fimbriae (*fimABCDEFGHI*) associated with urinary tract infection and biofilm formation were moderately to highly prevalent in the STs under study, except ST167. Although ST131 is known to be the most prevalent ST in bloodstream infections, ST73 was observed to harbor more virulence-related genes and toxins than ST131. A similar trend was also observed by Miajlovic et al. (54), which is in agreement with our findings. The *pap* (pyelonephritis-associated pilus) gene cluster, encoding P fimbriae, and *tcpC*, gene encoding immunomodulatory protein, were seen to be moderate to highly prevalent exclusively in the STs from phylogroup B2, such as ST12, ST127, ST73, and ST95. These gene clusters were previously reported to be present in the pyelonephritis- and urosepsis-related isolates (55).

As a result of the enormous amount of information available about the genomic features, classical analysis methods proved inadequate for identifying associations among different STs. Therefore, we adopted the machine learning approach, in which classification algorithms that support multiclass classification, natively, were hypertuned and the ST-associated features were identified using feature selection methods. Among the 435 relevant features identified to be important by the best-performing model, we tried to deduce the biological significance of a total of 86 proteins that showed differential prevalence among different STs (Fig. 6). Further analysis of these proteins, including those with unknown functions, which were observed to be present exclusively in either a single ST or STs from the same phylogroup, could help in identifying ST-specific or phylogroup-specific signatures. One of the toxin-antitoxin proteins, MazE-MazF, linked to programmed cell death under stress conditions (56), was notably missing from ST131, ST69, ST117, ST12, and ST73 genomes. This observation is in agreement with our previous study wherein we reported the absence of MazE-MazF from ST131 genomes (16). An overall abundance of toxin-antitoxin genes was associated with STs from certain phylogroups, such as A, D, E, and F. A lesser abundance of the identified toxin-antitoxin genes was observed in phylogroup B2 compared to that of other phylogroups, which is in agreement with a previous study by Fiedoruk et al. (57). Previous studies have linked plasmid stabilization with toxin-antitoxin loci, which help bacteria attain dormancy, and such bacteria are known as persisters. These persisters are observed to attain tolerance to antibiotics even in the absence of AMR genes (58). Further large-scale phylogenomic studies with antibiotic resistance phenotype information can demonstrate the evolution of toxins and their antidote with respect to ST/phylogroup and their association with antimicrobial resistance.

One of the limitations of this study was the inability to capture the multicollinearity among the genomic features, prior to modeling. Thus, the important genomic features identified by the RF model could only be representing clusters of collinear features. Further, analyzing the clusters of collinear features using unsupervised methods followed by supervised learning classification may result in better performance of the model and help in capturing additional salient features.

In conclusion, this study could identify critical genomic features with varied prevalence patterns among 19 different ST lineages with the help of supervised machine learning methods. These genomic features could possibly be involved in the evolution and spread of the STs in clonal complexes with high risk of transmission. Further in-depth analyses of these features with respect to their associated phenotypes might elucidate the patterns of acquisition of resistance, pathogenicity, and fitness among the individual clonal lineages.

## MATERIALS AND METHODS

### Collection and curation of genome data.

Total assembly information (complete and draft) of E. coli was downloaded from NCBI as of 12 February 2020, from which 14,295 genomes were shortlisted for downloading based on the criteria that each assembly has (i) fewer than 200 scaffolds/contigs, (ii) GC percentage between 48 and 51%, (iii) genome size ranging between 4.4 and 6 MB, and (iv) coding DNA sequence (CDS) greater than 2,000 bp. The associated biosample information and .xml files of these genomes were then downloaded from the NCBI Biosample database, which was further parsed and tabulated as metadata information using in-house python scripts. A total of 13,967 genomes with supporting biosample information were successfully downloaded. Quast (v.5.1.0) (59) was used to confirm the quality of these downloaded assemblies.

### Shortlisting of STs for comparative genome analysis.

STs of a total of 13,967 genomes were identified using an *in silico* MLST tool (https://github.com/tseemann/mlst) based on the PubMLST database (https://pubmlst.org/). Among the predicted STs, those representing at least 100 genomes (from a total database of 5,653 genomes, including 677 complete and 4,976 draft assemblies) and belonging to one or more of the 19 known STs were considered for further analysis. The minimum *N*50 value of the draft genome assemblies was found to be ∼50,000. Phylogroups of the respective genomes were identified using the EzClermont typing tool (v0.6.3) (60). The final selected genomes were observed to be from different parts of the world, collected from a wide range of isolation sources, hosts, and disease conditions, some representing high-risk clones with AMR, and were deposited in the NCBI database (includes missing biosample information). The metadata of the selected genomes, with their accession, biosample, ST, and phylogroup information, are provided in File S1 at https://github.com/Sabiha-NGS/EcoliGenomics2021.

### Profiling of resistome, virulome, and other important genomic features.

Screening of AMR genes and virulence genes across the 5,653 genomes was performed using ABRicate (https://github.com/tseemann/abricate) with an identity and query coverage of 75% against CARD (61) and VFDB (62) databases, respectively. Chromosomal point mutations associated with AMR were identified using the standalone version of the software PointFinder (v.4.1.0) (identity, 80%; query coverage, 60%) (63). Integrons and transposon regions from these genomes were identified using the standalone version of the consolidated tool BacAnt (v.3.3.1) with default parameters (64). Percent presence of each of these features (genes/mutations/integrons/transposons) at the ST level was calculated using the formula (presence in no. of genomes of ST/total no. of genomes in ST) × 100. Those features with at least 20% presence in any of the STs were plotted using matplotlib and seaborn libraries of Python.

Plasmid profiling and identification of serotypes of the 5,653 genomes were performed using PlasmidFinder (v.2.1) (65) and ECTyper (v.1.0) (66) with default parameters (https://github.com/phac-nml/ecoli_serotyping). Associations between serotypes and plasmid types with ST classes were calculated with the help of a chi-square test using the sklearn.feature_selection.chi2 module from Python, where 26 out of 31 plasmid classes and 301 out of 579 serotypes were found to be significantly (*P* ≤ 0.005) enriched.

### Generation of pangenome data.

The curated data set of 5,653 genomes was annotated with PROKKA (v.1.14.5) software (67), and the pangenome analysis was done by Roary (v.3.13.0) (68) with default parameters.

### Machine learning pipeline to perform classification and identify important features. (i) A subset of binary feature matrix for classification.

A binary feature matrix comprising the presence-absence status of 137,919 features from pangenome analysis, AMR genes, chromosomal point mutations, virulence-related genes, transposons, and integron classes were considered for the analysis. Given the large number of features, a subset of 11,988 features satisfying the following two conditions was then extracted for further analysis: (i) the feature should be present in more than 40% genomes in any one of the STs and (ii) the feature should be present in less than 90% of genomes in any one of the STs.

The selected subset of 11,988 features was tested for correlation with the target ST classes using the chi-square test.

### (ii) PCoA to understand how the genomes cluster based on a distance matrix.

PCoA of the subset binary feature matrix of 11,988 features using the Jaccard distance measure was performed, and each strain was then represented in a scatterplot in terms of its top two ordination axes (Fig. 5). A PERMANOVA of the Jaccard distance matrix was also performed to understand the significance of these features in explaining the genomic variations at the level of ST. The analysis was performed using scikit-bio and scipy spatial distance modules from python.

### (iii) Selection of best-performing classifier(s) and further hypertuning to identify optimal parameters.

The data set with the selected subset of features was split into training and testing data sets using the stratified split feature of the train-test split from the sci-kit learn module (69). The classification was performed on the training data set and further evaluated with the help of the test data using five different supervised learning classification algorithms, including Extra Trees, Random Forest (RF), KNeighbors (KNN), Categorical Naive Bayes, and XGBoost (Table 2). The RF and Extra Trees algorithms performed the best, with a testing accuracy of 0.99, F1 score of 0.9994, recall value of 0.9994, and precision of 0.9994. Hypertuning of parameters for an RF classifier using GridsearchCV with a cross-validation of 10-fold on the training data was then performed using the following parameters: *{‘oob_score’:[True]*, *‘min_samples_split’: [2*, *5*, *10*, *15*, *100]*, *‘min_samples_leaf’: [1*, *2*, *5*, *10]*, *’bootstrap’: [True*, *False]*, *‘criterion’: [‘gini’*, *‘entropy’]}*. The best-performing RF model with the parameters *{'bootstrap': True*, *'criterion': 'entropy'*, *'min_samples_leaf': 1*, *'min_samples_split': 2*, *'oob_score': True}* was then used to test the performance on the test data.

### (iv) Identification of important features and further validation using KNN.

*SelectFromModel* from the *sklearn.feature_selection* module of Python was used to identify the important features from the best RF model, and the resulting feature importance scores were further validated for a drop in the accuracy of the model. A total of 435 features with a significant drop in accuracy were considered to be significant, and a mean decrease accuracy plot of the top 50 features was plotted (see Fig. S3 at https://github.com/Sabiha-NGS/EcoliGenomics2021). To further validate the significance of these selected features, three KNN classifiers (with all features, important features, and without the important features) were trained and tested using the same training and testing data set. Classification reports of all three models are provided in File S5.

### Biological significance of the important genomic features identified by the best model.

Protein sequences of important features identified using the above-described analysis were further validated at the complete data set level using DIAMOND (v.2.0.13) (30). Only proteins with more than 70% identity and query coverage were given the status of present. The % presence of each of these protein sequences at the ST level was calculated using the formula (presence in no. of genomes of ST/total no. of genomes in ST) × 100. A total of 86 proteins satisfying the criteria used for feature selection were selected and compared visually using a cluster map. These 86 proteins were further annotated for their CDD using NCBI CD-Search with an expected threshold of 0.001 (70). Protein sequences of these 86 features are provided as File S7 (at https://github.com/Sabiha-NGS/EcoliGenomics2021).
